# Prognostic value of quantitative ctDNA levels in non small cell lung cancer patients

**DOI:** 10.18632/oncotarget.22470

**Published:** 2017-11-16

**Authors:** Mariano Provencio, María Torrente, Virginia Calvo, David Pérez-Callejo, Lourdes Gutiérrez, Fernando Franco, Clara Pérez-Barrios, Miguel Barquín, Ana Royuela, Francisco García-García, Coralia Bueno, Aranzazu Garcia-Grande, Carlos Camps, Bartomeu Massuti, Eduardo Sotomayor, Atocha Romero

**Affiliations:** ^1^ Medical Oncology Department, Hospital Universitario Puerta de Hierro-Majadahonda, Majadahonda, Spain; ^2^ Liquid Biopsy Laboratory, Biomedical Sciences Research Institute Puerta de Hierro-Majadahonda University Hospital, Majadahonda, Spain; ^3^ Biostatistics Department, Biomedical Sciences Research Institute Puerta de Hierro-Majadahonda University Hospital, Majadahonda, Spain; ^4^ Computational Genomics, Centro de Investigación Príncipe Felipe, Valencia, Spain; ^5^ Medical Oncology Department, Hospital Infanta Cristina, Parla, Spain; ^6^ Cytometry Unit, Biomedical Sciences Research Institute Puerta de Hierro-Majadahonda, Spain; ^7^ Medical Oncology Department, Hospital General de Valencia, CIBERONC Network, Valencia, Spain; ^8^ Medical Oncology Department, Hospital Universitario de Alicante, Alicante, Spain; ^9^ George Washington Cancer Center, Washington D.C., United States of America

**Keywords:** ctDNA, non-small cell lung cancer, tyrosine kinase inhibitor, EGFR

## Abstract

**Background:**

Circulating tumor DNA (ctDNA) levels correlate well with tumor bulk. In this paper we aim to estimate the prognostic value of the dynamic quantification of ctDNA levels.

**Materials and Methods:**

A total of 251 serial plasma samples from 41 non-small-cell lung cancer patients who carried an activating EGFR mutation were analysed by digital PCR. For survival analysis, ctDNA levels were computed as a time-dependent covariate.

**Results:**

Dynamic ctDNA measurements had prognostic significance (hazard ratio for overall survival and progression free survival according to p.T790M mutant allele frequency; 2.676 and 2.71 respectively; *P* < 0.05). In the same way, patients with p.T790M-negative or unchanging or decreasing plasma levels of sensitizing EGFR mutation were 12 and 4.8 times more likely to maintain response or stable disease, respectively, than patients in which the opposite occurred (*P* < 0.05).On average, the p.T790M mutation was detected in plasma 51 days before the assessment of progression disease by CT-scan. Finally, ctDNA outperformed CTCs for assessing tumor progression (*P =* 0.021).

**Conclusions:**

The appearance or increase in a unit of the p.T790M allele frequency almost triples the risk of death and progression. This information can be used to design clinical trials aiming to estimate whether T790M positive patients should start second line treatment based on molecular data rather than imaging data.

## INTRODUCTION

Molecular profiling of solid tumours via blood samples is an expanding field that has attracted important attention among medical oncologists. This approach enables clinicians to repeatedly interrogate the dynamic evolution of tumors due to the non-invasive nature of the procedure. Importantly, circulating tumor DNA (ctDNA) has proven to be an adequate source for biomarker testing and tumour bulk monitoring, suggesting its utility for treatment outcome monitoring. [1–5]. Specifically, in *EGFR* positive lung cancer patients treated with tyrosine kinase inhibitors (TKI) ctDNA has shown reliable correlations with tumor load and changes in response to treatment [3, 6–9], indicating a potential utility of this approach in the clinical management of NSCLC.

Importantly, it has been demonstrated that p.T790M can be effectively detected in plasma samples several months before disease progression is ascertained by radiologist [6]. It is not yet clear when should second line TKI treatments be started. At present, disease progression in T790M positive patients can be defined based on molecular data, imaging data and the clinical situation of the patient. Whether different patient’s management may impact on survival has not been explore yet.

In this prospective study, we assess the dynamic changes in *EGFR* mutation in plasma using an array-based digital PCR (dPCR) methodology. Plasma samples from NSCLC patients harboring activating *EGFR* mutations and treated with *EGFR* TKIs were longitudinally collected in order to evaluate the prognostic value of the dynamic quantification of ctDNA levels and its potential utility in daily clinical practice.

## RESULTS

### Study cohort

This study reports daily clinical practice data obtained from 41 lung cancer patients that were prospectively enrolled between February and December 2015, from whom 251 serial plasma samples were obtained and analyzed during a median follow-up of 10 months. Routine follow-up examinations were performed by a medical oncologist every 3 weeks for the first 3 months, and every 12 weeks thereafter or as required according to the oncologist’s criteria. The pathological characteristics of the study population are summarized in Supplementary Table 1.

An average of 6.1 cfDNA samples were analysed per patient. cfDNA from all blood samples was analysed for the amount of the original sensitizing *EGFR* mutation as well as the p.T790M mutation. Overall fluctuations in ctDNA plasma levels correlated with tumor response ascertained by radiologist (Supplementary Figure 1).

### Sensitizing and p.T790m *EGFR* mutations tracking to monitor treatment outcome

dPCR was performed in all collected plasma samples (*N =* 251). ctDNA was detected in 36 (88%) patients. Among patients in whom ctDNA was not detected, three cases corresponded to patients in complete response (CR). As expected, the *EGFR* sensitizing mutation identified originally in the tumor sample was also detected in all the baseline plasma samples (treatment naïve patients). The p.T790M resistance mutation was never detected at baseline.

During the study follow up, 15 deaths were recorded and progressive disease (PD) was observed in 26 patients (63%), of whom 22 were undergoing first-line treatment while four of them second-line. In all cases, a plasma sample was obtained upon PD. A re-biopsy of the tumour lesion was only performed in seven cases (28% of the patients that had progressed). Of these, the p.T790M mutation was detected in three tumor samples. In these three cases, p.T790M was also detected in the matched plasma sample.

The median progression free survival (PFS) to first-line TKI treatment was 14.2 months (95% CI 8.0–23.9). Median PFS in patients with tumors carrying an exon 19 deletion was 23.9 months. These patients showed significantly increased PFS (HR = 0.29; 95% CI = 0.13–0.69; *P = 0.005*). In contrast, patients with tumors carrying an insertion in exon 20 exhibited worse outcomes (median PFS = 2.4 months) (Supplementary Table 2). No significant differences were observed with respect to treatment.

Our results consistently showed that the detection of the p.T790M resistance mutation as well as an increment in the quantification of the original sensitizing *EGFR* mutation in serial plasma samples was associated with the assessment of PD (*P* < 0.001). The appearance of the p.T790M mutation always occurred together with an increase in the amount of sensitizing *EGFR* mutation. Specifically, an increase of the original sensitizing *EGFR* mutation was displayed in 24 (92%) out of 26 patients in which PD was observed during the study follow up. In addition, in 18 of these cases (69%) the p.T790M mutation was concomitantly detected (Table 1). According to our data, patients with p.T790M-negative plasma over the course of treatment were twelve times more likely to maintain response or stable disease than those patients in which p.T790M mutation was detected in at least one plasma sample. Similarly, when sensitizing *EGFR* plasma levels did not increase, patients were almost five (4.8) times less likely to have a PD than patients in which sensitizing *EGFR* plasma levels increased.

**Table 1:** Number of patients showing an increase of the sensitizing mutation or the appearance of p.T790M mutation according to tumor response (Progressive disease vs others)

Patient outcome	*N*	Positive plasma T790M (*N*)	*P*	Increase in EGFR sensitizing mutation	*P*
Progressive disease	26	18	< 0.001	24	< 0.001
Other	15	1		5	

As shown, T790M was detected in the tumor of one patient who had progressed after first-line treatment. However, this patient responded to a second-line TKI and did not progress during the study follow-up period.

**Figure 1:**
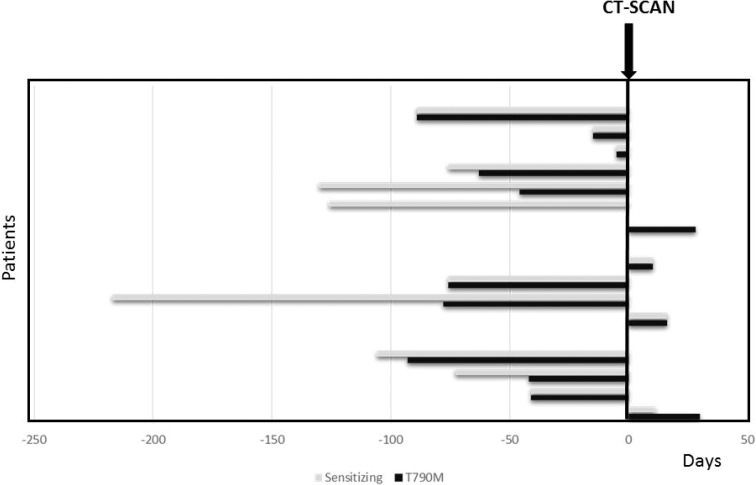
Number of days since the earliest identification of the T790M mutation (black bars) and earliest identification of an increment of sensitizing mutation (grey bars) in the blood and assessment of disease progression by CT-scan The figure shows how many days earlier or later ctDNA could be detected before or after tumour progression was ascertained by imaging.

On average, the p.T790M mutation was detected in plasma 51 days before the assessment of PD by CT-scan. Specifically, in 50% of these patients the p.T790M mutation was identified in blood within the same two weeks in which PD was detected. In 44% of these patients, p.T790M was detected in blood between 41 and 93 days before PD was evident according to RECIST criteria (Figure 1) and in one case, p.T790M was identified in blood after PD was assessed (Figure 1). An increase of the sensitizing mutation occurred before the detection of p.T790M in blood samples in 39% of the patients, as shown in Figure 1.

We similarly detected a substantial decrease of p.T790M and the sensitizing mutation as a surrogate biomarker for tumor response in all patients treated with osimertinib (*N* = 6)

Finally, we evaluated the prognostic value of ctDNA quantification using a Cox proportional hazards model. As presented in Table 2, increasing levels in sensitizing and p.T790M mutation were associated with inferior PFS during first-line TKI treatment (*P* < 0.05), indicating that ctDNA quantification is informative in terms of prognosis. As shown, one percentage point increase in p.T790M mutant allele frequency (MAF) approximately triples (2.7 times) the risk of PD or death.

**Table 2:** Multivariate Cox proportional hazards analyses and 95% confidence interval of the four explanatory variables

	OS			PFS		
Variable	HR	95% CI	*P*	HR	95% CI	*P*
T790M allele fraction	2.676	1.384–5.176	0.003	2.705	1.18–3.92	0.001
Sensitizing allele fraction	1.047	0.931-1.104	0.088	1.083	1.01-1.11	< 0.001

### Comparison of circulating tumor DNA and circulating tumor cells (CTCs) as prognostic biomarkers

In order to evaluate the accuracy of each methodology (ctDNA and CTCs) to discriminate between progressed and non-progressed patients, according to RECIST v1.1 criteria, sensitivity, specificity, positive likelihood ratio and negative likelihood ratio were calculated. For CTCs analyses, CTCs counts were available across 70 serial time points for 29 patients. The same subset of patients was used to evaluate ctDNA. CTCs cut-offs of < 5 vs ≥ 5 were established. With respect to ctDNA quantification, molecular progression was defined as an elevation in sensitizing *EGFR* mutation quantification or appearance of the T790M mutation in two or more consecutive samples with the last sample being obtained at the time of progression. As shown in Table 3, ctDNA showed improved sensitivity, specificity, positive likelihood ratio and negative likelihood ratio than CTCs for assessing tumor progression (*P = 0.021*).

**Table 3:** Sensitivity, specificity, positive likelihood ratio and negative likelihood ratio of ctDNA and CTCs for assessing tumor progression (*N* = 70)

	ctDNA		CTCs	
		95 CI		95 CI
Sensitivity	94.4	73–100	44.4	21–69
Specificity	72.7	39–94	63.6	31–89
Positive	3.5	1.31–9.15	1.2	0.48–3.12
Negative	0.1	0.01–0.53	0.9	0.48–1.6

### Quantitative performance of EGFR assays

ctDNA fluctuations measured as mutated copies/ml or as the ratio mutant allele fraction showed similar patterns. According to our data, in positive samples the ratio of mutant DNA molecules vs total DNA molecules ranged from 0.10% to 38.25% and the number of mutated copies in positive samples ranged from 144 to 727562.5 copies/ml.

For sensitivity assays, cell lines carrying exon p.E746_A750delELREA (c.2236_2250del15), p.G719S (c.2155G > A), p.T790M (c.2369C > T) and p.L858R (c.2573T > G) mutations were mixed at different allele concentrations with *wild type* (wt) DNA extracted from peripheral blood cells obtained from healthy donors. MAF correlated with their expected frequencies in all assays (Pearson’s correlation coefficient, 0.9782, 0.9991, 0.9954 and 0.9966 respectively; Supplementary Table 3). Limit of detection (LOD) for p.E746_A750delELREA (c.2236_2250del15), p.G719S (c.2155G > A), p.T790M (c.2369C > T) and p.L858R (c.2573T > G) assays were 0.15%, 0.04%, 0.08% and 0.07%, respectively. LODs were estimated for samples with an average of 300 copies/ml of wt DNA. Additionally, 10 wt cfDNA from healthy donors were used to evaluate the false positive signals. The Limit of quantitation (LOQ) was 0.01% for p.E746_A750delELREA assay, 0.00% for p.G719S assay, 0.01% for p.T790M and 0.00% for p.L858R assay. LOBs were estimated for samples with an average of 3000 copies/µl.

## DISCUSSION

Several previous studies have reported the feasibility of detecting resistance and sensitizing *EGFR* mutations in plasma samples and the early prediction of tumor progression compared to CT-scans using liquid biopsy [1, 3, 6–9]. However, there is a lack of studies reporting the clinical utility of liquid biopsies in the real world and while the usefulness of liquid biopsy has been evaluated in several clinical studies the real-world setting remains scarcely explored. In the real world, liquid biopsies may be collected differently than in clinical trials. Patients included in clinical studies may be significantly different from the general patient population. Moreover, it is well known that monitoring procedures in clinical studies can differ substantially from the routine clinical practice. Our study was aimed at clinical validation, integrating dPCR technology in current oncology. For this purpose, the extraction of samples was performed as indicated by the medical oncologist during the routine follow up of the patients. Our data present a high number of extractions per patient (up to 19 samples/patient), with an average of 6 samples/patient making a total of 251 samples being prospectively collected.

As previously reported [6–9], this study shows that the detection of the T790M resistance mutation in blood, together with an increase of the original sensitizing *EGFR* mutation in serial plasma samples, was associated with the diagnosis of PD (*P* < 0.005). Remarkably, in 39% of patients an increase of the sensitizing mutation occurred prior to the detection of T790M in blood samples.

The appearance in the p.T790M mutation occurred at a mean of 51 days before progression was radiologically assessed, and it was accompanied by an increase of the sensitizing mutation. At present, the duration of TKI therapy still relies on the oncologist´s expertise and frequently patients continue therapy beyond progression. Molecular progression might occur even before radiological disease progression [1, 6, 10]; raising the recurrent question of what is the optimal duration for TKI first line treatment.

Cox proportional hazard models showed that increasing levels of sensitizing and p.T790M mutation were associated with shorter PFS (*P* < 0.005 in all cases), indicating that ctDNA quantification is informative in terms of prognosis. In addition, one percentage point increase in p.T790M MAF approximately triples (2.7 times) the risk of disease progression to first line treatment or death. It is important to point out that unlike other series [11] HR were estimated considering ctDNA levels as a time dependent co-variate in our study.

However, other researchers [12] failed to demonstrate any significant differences in PFS with respect to baseline ctDNA measurements. A single determination at a specific time might not yield sufficient information, and therefore the dynamic quantification of ctDNA should be emphasized. As a matter of fact, ctDNA should be regarded as a time dependent variable.

When sensitizing *EGFR* plasma levels did not increase, patients were approximately five (4.8) times less likely to have PD than those patients in which sensitizing *EGFR* plasma levels increased. Remarkably, patients with p.T790M-negative plasma over time were twelve times more likely to maintain response or SD than those patients in which a p.T790M mutation was detected in at least one plasma sample. Decision-making in asymptomatic clinical or radiological progressions that present little threat to a patient’s life is still a challenge. Until now, evidence of the clinical management of these patients derives from clinical studies that did not include or analyse prognostic molecular parameters [13, 14], unlike the results presented in this work that could be used to plan clinical trials to validate the real clinical benefit of selection of treatment based on liquid biopsy molecular data.

## MATERIALS AND METHODS

### Study population and data management

A total of 251 serial plasma samples were collected from 41 NSCLC patients who were prospectively enrolled between February and December 2015. The median follow-up was 19 months. Written informed consent was obtained from every patient. Eligible patients were male and female patients with a pathologically confirmed diagnosis of stage IIIB-IV NSCLC tumor with an *EGFR* mutation in primary tumor tissue. A complete staging workup was performed prior to recruitment. Blood samples were collected as follows: at diagnosis, during follow up evaluations and when appointed by the medical oncologist if further determination of clinical status, radiological assessments, toxicity events or even TKI dose reduction may be needed. Demographic characteristics, clinicopathological features, tumor mutational status, vital status, disease status, drug dose adjustments or discontinuation of medication were collected in the study electronic database.

The protocol was approved by the Hospital Puerta de Hierro Ethics Committee (internal code PI/144–14) and was conducted in accordance with the precepts of the Code of Ethics of The World Medical Association (Declaration of Helsinki).

### Laboratory procedures

For ctDNA analysis, peripheral whole blood was collected from each subject in a 5 ml EDTA tube containing a gel barrier (PPT™, BECTON DICKINSON) to separate the plasma from blood cells after centrifugation. All samples were processed as previously described [5, 15]. cfDNA was extracted using a starting volume of 1 ml of plasma with a Maxwell® RSC instrument (Promega), using the Maxwell® RSC ccfDNA Plasma Kit, as specified by the manufacturer and was eluted in 50 µl of the supplied buffer. Germline DNA was obtained from blood leukocytes with a MagNA Pure LC total nucleic acid extraction kit in a MagNA Pure LC instrument (Roche Diagnostics, Penzberg, Germany). cfDNA samples were then analysed by dPCR using Rare Mutation Assays for p.T790M (AHRSROS), p.L858R (AHRSRSV), p.G719A (AHABH29), p.G719C (AH0JEWC), p.G719S (AHZAGP4), p.H773_V774insH (AH5I7PA), p.D770_N771insG (AH7031Q), p.L747_T751 > P (AHFA92K), p.L747_A750 > P (AHS1PY0), p.E746_T751 > A (AHHS6E0), p.E746_A750delELREA (AHLJ0XO), p.L747_T751delLREAT (AHCTDP3) and p.L747_S752delLREATS (AHGJ78R ) on a QuantStudio® 3D Digital PCR System (Applied Biosystems, South San Francisco, CA), as previously described [5]. Samples were considered positive when the mutant allele fraction (MAF) was greater than the limit of detection assessed for individual assays (Supplementary Table 3). A wt control DNA was included in every run.

For CTC analysis, blood was collected in CellSave Preservative Tubes (Veridex). Pre-enrichment of CTCs was performed by density gradient centrifugation. A double gradient was formed by layering 5 ml of HISTOPAQUE-1077 (Sigma Diagnostics, St. Louis, MO) over an equal volume of HISTOPAQUE-1119 (Sigma Diagnostics, St. Louis, MO). Blood samples were carefully layered onto the upper HISTOPAQUE-1077 medium. The tubes were then centrifuged at 700g for 30 min. The granulocyte cell fraction was found at the 1077/1119 interphase, whereas the mononuclear cell fraction was found at the plasma/1077 interphase. Both cell fractions were mixed and washed with 10 ml of PBS and centrifuged at 300g for 10 minutes.

Circulating tumor cells (CTCs) enrichment was performed using selective positive immunomagnetic cell separation, with EpCAM microbeads (Miltenyi-Biotec, Germany). The magnetically labeled cell suspension was then purified and enriched in a magnetic field using an AutoMACS (Miltenyi Biotec) magnetic separator. After capture and immunomagnetic enrichment, fluorescent reagents were added for intracellular and extracellular phenotypic identification of CTCs by flow cytometry. Cells were fluorescently labeled with anti-human CD45-APC (Clone: 5B1), anti-human CD326-Epcam PE (Clone: HEA-125), a nuclear dye and anti-Cytokeratin-FITC (CK3–6H5) (Miltenyi Biotec) antibodies. Samples were analyzed by flow cytometry on a MACSQuant Analyzer (Miltenyi-Biotec) equipped with three solid-state air-cooled lasers which allow simultaneous detection of 10 parameters. The Flow cytometry dot-plots were generated by logarithmic amplification of fluorescence emitted by single viable cells. The three channels of fluorescence were acquired sequentially with the following excitation and emission parameters: (488 nm, 500–540 nm) for cytokeratin signal, (546 nm, 557–572 nm) for EpCAM, and (633 nm, 645–750 nm) for CD45. Data analyses were performed using sensitive MACSQuantify^™^ Software (Miltenyi Biotec).

### Tumor response evaluation

Computed tomography (CT) measurements and magnetic resonance imaging (MRI) were obtained as clinically indicated. The clinical response was evaluated according to RECIST criteria v1.1 combined with a blinded medical judgment of benefits from the treatment. Additionally, whole body 18F-fluoro-2-deoxy-D-glucose-positron emission tomography (18FDG-PET) CT scans were performed as clinically indicated using a Siemens Biograph 6 True Point PET-CT (Siemens). A 350–450MBq 18F-FDG dose was administered 55–65 min before image acquisition. Reconstruction was performed using an iterative method and attenuation/scatter correction.

### Statistical analysis

The association between categorical variables was tested by Fisher’s exact test. PFS was defined as the time between the date the patient was enrolled in the study and the date when tumor progression was diagnosed or death from any cause.

Survival analysis was performed by fitting a different Cox regression model for each of the variables: p.T790M allele fraction, sensitizing allele fraction, number of p.T790M copies/ml and number of sensitizing mutation copies/ml. In order to facilitate interpretation of HR, p.T790M copies/ml and number of sensitizing mutation copies/ml were transformed to log2. Predictors were modelled as continuous time-dependent covariates using tools for creating time-dependent covariates in R (https://cran.r-project.org/web/packages/survival/vignettes/timedep.pdf) [16]. A value of *P* < 0.05 was considered statistically significant. Statistical analyses were performed using Stata 14.1 and R 3.1.2 software.

## SUPPLEMENTARY MATERIALS FIGURE AND TABLES




