# Supplementary material for: Prognostic value of quantitative ctDNA levels in non small cell lung cancer patients
Source: Oncotarget. 2017 Nov 16;9(1):488–94. doi: 10.18632/oncotarget.22470 (PMC5787483; doi:10.18632/oncotarget.22470)
Supplement: Supplementary file 1 [file oncotarget-09-488-s001.pdf]

Prognostic value of quantitative ctDNA levels in non small cell lung cancer patients

SUPPLEMENTARY MATERIALS

Supplementary Table 1: Clinicopathological features of the study population

Median age at diagnosis	61 years (30–83)	*N*	%
**Sex**	Women	23	56%
	Men	18	44%
**Smoking status**	Former/quit	19	46%
	Never	22	54%
**Histology**	Adenocarcinoma	39	95%
	Squamous cell carcinoma	2	5%
**UICC Stage**	III	4	10%
	IV	37	90%
**EGFR mutation**	exon 19 deletion	24	59%
	exon 20 insertion	3	7%
	G719X	4	10%
	L858R	10	24%
**Number of treatment lines monitored**	1 line	32	78%
**throughout the study**	2 lines	6	15%
	3 lines	3	7%
**Treatment1**	1st line TKI treatment	35	85%
	Gefitinib	11	27%
	Erlotinib	20	49%
	Afatinib	5	12%
	Gefitinib + Olaparib	1	2%
	2nd line TKI treatment	4	10%
	Osimertinib	1	2%
	Afatinib + Cetuximax	1	2%
	Erlotinib + Pemetrexed	1	2%
	Gefitinib + Pemetrexed	1	2%
	Non TKI treatment (stage III patients)	2	5%
	Cisplatine-Vinorelbine	1	5%
	Pemetrexed	1	5%

[1]Initial treatment within the study period.

Supplementary Table 2: Progression free survival (PFS) and 95% confidence interval (CI) according to sensitizing mutation

Tumor Mutation	median PFS (in months)	95% CI
deletion exon 19	23.9	8.7–58
G719X	3.13	NA
L858R	12.8	5.4–18.6
insertion exon 20	2.4	NA
Overall	14.2	8.0–23.9

Supplementary Table 3: Limit of detection (LOD) and limit of quantification (LOQ) estimation for rare allele assays according to ICH guidelines. See_Supplementary_Table 3

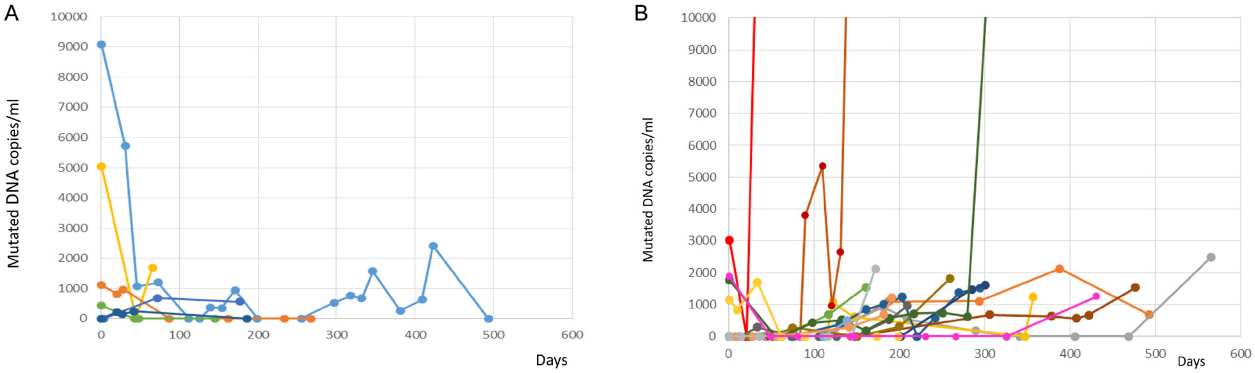

Supplementary Figure 1: (A) and (B) Performance of EGFR sensitizing mutation assays among non-progressed patients during the study follow-up and had a partial response as their best response (as defined by RECIST criteria). Only patients with a minimum of four plasma samples extracted at different time points are presented (N = 6). As shown in the figure, patients experienced a decrease in EGFR-activating mutation levels. In responding patients plasma EGFR mutations levels fluctuated as much as 9076 copies/ml to undetectable levels. Only patients with a minimum of four plasma samples extracted at different time points are presented (N = 17).
