# Supplementary material for: Prognostic value of quantitative ctDNA levels in non small cell lung cancer patients
Source: Oncotarget. 2017 Nov 16;9(1):488–94. doi: 10.18632/oncotarget.22470 (PMC5787483; doi:10.18632/oncotarget.22470)
Supplement: Supplementary file 2 [file oncotarget-09-488-s002.docx]

**Supplementary Table 3: Limit of detection (LOD) and limit of quantification (LOQ) estimation for rare allele assays according to ICH guidelines.**

**p.T790M**

**
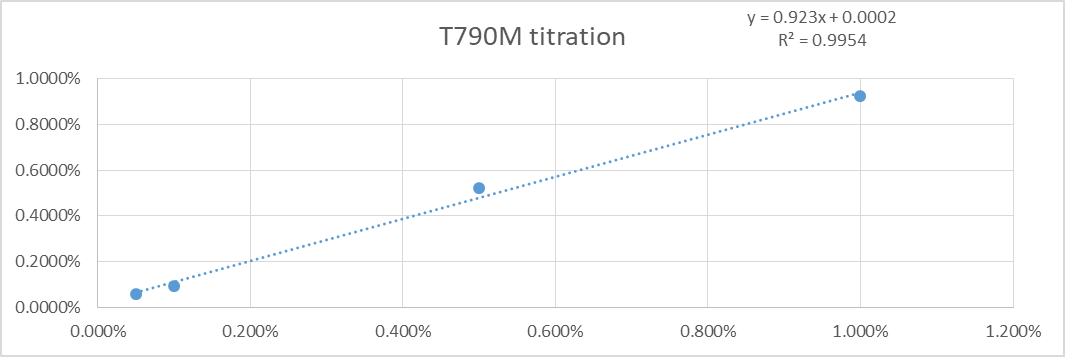
**

**p.L858R**

**
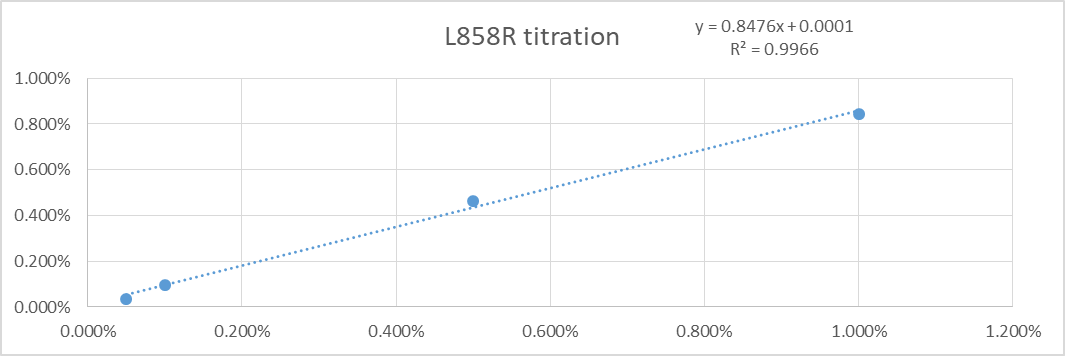
**

**p.G719S**

**
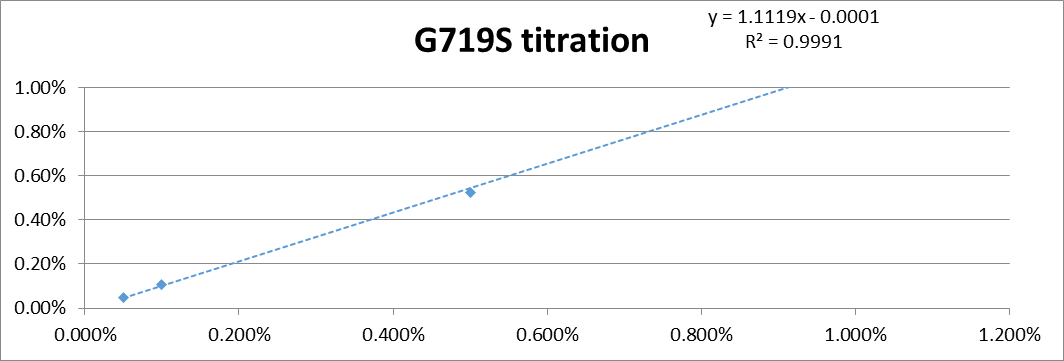
**

**p.E746_A750delELREA**

**
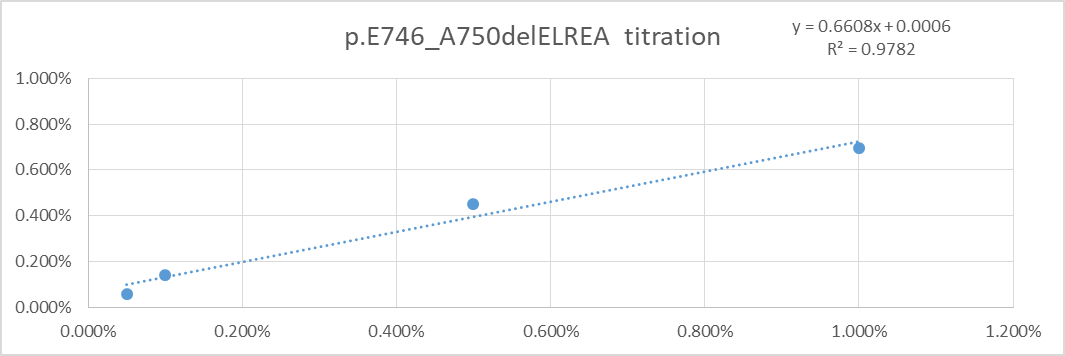
**
